# Supplementary material for: Reconciling Mining with the Conservation of Cave Biodiversity: A Quantitative Baseline to Help Establish Conservation Priorities
Source: PLoS One. 2016 Dec 20;11(12):e0168348. doi: 10.1371/journal.pone.0168348 (PMC5173368; doi:10.1371/journal.pone.0168348)
Supplement: S1 Dataset — (ZIP) [file pone.0168348.s002.zip › Taxa/Serra Norte/SN_2013/N4WS/Lista N4WS-01.pdf]

CAVIDADE N4WS-0001

Classe	Ordem	Fam/Outros	Gên/Outros	Espécie	S-U	U-S	Zon.
Arachnida	Acari	Argasidae	*Ornithodoros*	sp.1	X	X	P
Arachnida	Acari	Laelapidae		sp.1	X		P
Arachnida	Acari	Laelapidae		sp.2	X		P
Arachnida	Acari	Macrochelidae		sp.2	X		P
Arachnida	Acari	Ologamasidae		sp.1		X	E
Arachnida	Acari	Otopheidomenidae		sp.1		X	P
Arachnida	Acari	Podocinidae	*Podocinum*	sp.1	X		P
Arachnida	Acari	Rhagidiidae		sp.1		X	P
Arachnida	Araneae	Ctenidae		jovem		2	P
Arachnida	Araneae	Drymusidae		jovem	X		P
Arachnida	Araneae	Ochyroceratidae	*Speocera*	sp.1	X	X	P
Arachnida	Araneae	Pholcidae	*Mesabolivar*	*eberhard*	X	X	P
Arachnida	Araneae	Prodidomidae	*Lygromma*	sp.1		X	P
Arachnida	Araneae	Theridiidae		jovem	X		P
Arachnida	Araneae	Theridiosomatidae	*Plato*	sp.1	X	X	E/P
Arachnida	Araneae	Theridiosomatidae	*Plato*	sp.3		X	E
Arachnida	Pseudoscorpiones	Chernetidae	*Spelaeochernes*	sp.1	X	X	E/P
Arachnida	Pseudoscorpiones	Chthoniidae	*Pseudochthonius*	sp.1	X		P
Arachnida	Schizomida	Hubbardiidae	aff. *Rowlandius*	jovem		X	E
Chilopoda	Scolopendromorpha	Scolopendridae	*Cormocephalus*	sp.1		1	E
Chilopoda	Scolopendromorpha	Scolopocryptopidae	*Scolopocryptops*	sp.1	1		P
Diplopoda	Polydesmida	Fuhrmannodesmidae		sp.1	X		P
Diplopoda	Polydesmida	Pyrgodesmidae		sp.3	X		P
Diplopoda	Polyxenida	Hypogexenidae		sp.1	X	X	P
Entognatha	Collembola	Isotomidae		sp.1	X	X	E/P
Entognatha	Collembola	Paronellidae		sp.1		X	E/P
Entognatha	Diplura	Campodeidae		sp.1	X	X	E/P
Entognatha	Diplura	Projapygidae		sp.1		X	E
Insecta	Blattodea	Polyphagidae		jovem	2	1	E/P
Insecta	Coleoptera	Elateridae		jovem	X		P
Insecta	Coleoptera	Tenebrionidae		jovem		X	E
Insecta	Diptera	Canacidae		jovem	X		P
Insecta	Diptera	Cecidomyiidae	Cecidomyiinae	sp.	X	X	E/P
Insecta	Diptera	Ceratopogonidae		jovem		X	P
Insecta	Diptera	Chironomidae		sp.1	X		P
Insecta	Diptera	Psychodidae	*Sciopemyia*	*sordellii*		X	E/P
Insecta	Diptera	Sciaridae	*Epidapus*	sp.	X		P
Insecta	Diptera	Tipulidae	Tipulinae	sp.	X	X	E/P
Insecta	Heteroptera	Cydnidae	*Pangaeus*	sp.1	X	X	E/P
Insecta	Heteroptera	Reduviidae	Emesinae	sp.1	1	1	P
Insecta	Heteroptera	Reduviidae	Reduviinae	jovem	7	2	E/P
Insecta	Heteroptera	Schizopteridae	Schizopterinae	sp.1	X		P
Insecta	Homoptera	Cixiidae		jovem		X	E
Insecta	Hymenoptera	Formicidae	*Camponotus*	sp.1	X		P
Insecta	Hymenoptera	Formicidae	*Nylanderia*	sp.1	X		P
Insecta	Hymenoptera	Formicidae	*Nylanderia*	sp.3		X	E/P
Insecta	Hymenoptera	Formicidae	*Pheidole*	sp.2	X		P
Insecta	Hymenoptera	Formicidae	*Solenopsis*	sp.1	X		P
Insecta	Isoptera	Termitidae	*Nasutitermes*	sp.	X	X	E/P

Insecta	Lepidoptera	Gelechioidea		sp.3		X	E
Insecta	Lepidoptera	Noctuoidea		sp.1	1		P
Insecta	Neuroptera	Myrmeleontidae		jovem	1	1	P
Insecta	Orthoptera	Phalangopsidae	*Phalangopsis*	sp.	21	7	P
Insecta	Psocoptera	Psocomorpha		jovem		X	E
Insecta	Psocoptera	Psyllipsocidae		jovem	X	X	E/P
Insecta	Thysanura	Nicoletiidae		sp.1	X		P
Gastropoda	Pulmonata	Subulinidae	*Lamellaxis*	sp.	X		P
Amphibia	Anura	Strabomantidae	*Pristimantis*	cf. *fenestratus*	1	1	E/P
Mammalia	Chiroptera	Emballonuridae	*Peropteryx*	sp.	1		P
